# Virucidal Influence of Ionic Liquids on Phages P100 and MS2

**DOI:** 10.3389/fmicb.2017.01608

**Published:** 2017-08-24

**Authors:** Susanne Fister, Patrick Mester, Julia Sommer, Anna K. Witte, Roland Kalb, Martin Wagner, Peter Rossmanith

**Affiliations:** ^1^Christian Doppler Laboratory for Monitoring of Microbial Contaminants, Institute for Milk Hygiene, Milk Technology and Food Science, Department for Farm Animals and Public Veterinary Health, University of Veterinary Medicine Vienna, Austria; ^2^Proionic Production of Ionic Substances GmbH Grambach, Austria; ^3^Institute for Milk Hygiene, Milk Technology and Food Science, Department for Farm Animals and Public Veterinary Health, University of Veterinary Medicine Vienna, Austria

**Keywords:** ionic liquids, virus, phage, side chain effect, structure activity relationship, disinfection

## Abstract

An increasing number of publications describe the potential of ionic liquids (ILs) as novel antimicrobials, antibacterial coatings and even as active pharmaceutical ingredients. Nevertheless, a major research area, notably their impact on viruses, has so far been neglected. Consequently the aim of this study was to examine the effects of ILs on the infectivity of viruses. A systematic analysis to investigate the effects of defined structural elements of ILs on virus activity was performed using 55 ILs. All structure activity relationships (SARs) were tested on the human norovirus surrogate phage MS2 and phage P100 representing non-enveloped DNA viruses. Results demonstrate that IL SAR conclusions, established for prokaryotes and eukaryotes, are not readily applicable to the examined phages. A virus-type-dependent IL influence was also apparent. Overall, four ILs, covering different structural elements, were found to reduce phage P100 infectivity by ≥4 log_10_ units, indicating a virucidal effect, whereas the highest reduction for phage MS2 was about 3 log_10_ units. Results indicate that future applications of ILs as virucidal agents will require development of novel SARs and the obtained results serve as a good starting point for future studies.

## Introduction

Ionic liquids (ILs) continue to find numerous applications, ranging from simple solvents to tools for chemical synthesis, CO_2_ capture, coatings etc. (Thuy Pham et al., 2010). With the increasing range of IL applications from laboratory to factory scale, their toxicity and environmental fate have rightly been questioned over the past decade (Stolte et al., 2007; Bubalo et al., 2014). Past research has shown that IL toxicity is highly variable and ILs can be classified over a range from non-toxic (comparable with common organic solvents) to toxic (comparable to highly active biocides; Hough et al., 2007). In almost all investigated biological test systems (from enzyme inhibition assays to *in vitro* tests in vertebrates) several pronounced IL toxicity structure–activity relationships (SAR) have been identified (Thuy Pham et al., 2010). Although, ongoing investigations have highlighted some inconsistencies in these SAR findings (depending on the test system used), generally it is believed that it is the cation that mainly determines IL toxicity (Ranke et al., 2007; Stolte et al., 2007). One relationship from SAR studies that has been frequently reported relates increased IL toxicity with increased cationic side-chain length (until a certain threshold or “cut-off effect” is reached; Docherty and Kulpa, 2005). This side-chain effect most probably results from increased lipophilicity, which permits interaction with biological membranes (Santos et al., 2015). Side chain length is currently the most significant indicator of biological activity. In addition to the cationic side chain length, the head group has been investigated, but this does not distinctly influence IL toxicity (Stolte et al., 2007).

Although research for some time now has mainly focused on cation effects, it is becoming clear that the anion also contributes to IL toxicity. Anion toxicity is most pronounced in short-chained congeners, where cationic toxicity is least (Stolte et al., 2007; Ventura et al., 2012; Mester et al., 2015). It appears that the anion influences IL toxicity by increasing chaotropicity (Mester et al., 2012). Further, increasing the number of hydroxyl groups, which increases IL polarity, enhances general toxicity, presumably due to enhanced interaction with negatively charged biological membranes (Reichardt, 2005; Cho et al., 2009; Santos et al., 2015). Moreover, as with the cation moiety, elongated anionic alkyl chains can increase toxicity in some, but not all tested systems (Ventura et al., 2014; Santos et al., 2015). While fluorine-containing anions have been suggested to be relatively toxic, halogen anions in general do not follow this trend (Stolte et al., 2006; Cho et al., 2008).

With increasing numbers of publications on this subject, understanding, and predictability of IL toxicity has improved significantly over the past decade. This has led to the realization that their tuneable toxicity may be advantageous, permitting consideration as new antimicrobials, antibacterial coatings, antimycotic agents, and even as active pharmaceutical ingredients (Hough et al., 2007; Ferraz et al., 2011; Kemp, 2011; Coleman et al., 2012; Andreeá Cojocaru, 2013; Hartmann et al., 2016; Piotrowska et al., 2017). However, despite these proposed applications, it is clear that the potential impact of ILs on viruses has been neglected. Besides a serious study by Byrne et al. (2012), there has been only one other, notably from our working group (Fister et al., 2015) that has dealt with this theme. The former study concerned stabilization of the tobacco mosaic virus with protic ILs. This is a virus that is an important genetic vector in plant biotechnology (Byrne et al., 2012). These investigators reported enhanced stabilization of the virus stored in ethyl ammonium and diethyl ammonium mesylate, while storage in triethyl ammonium and tripropyl ammonium mesylate effected a change in the secondary structure of the virus particles. In the latter study we established an IL-based method for destabilization of virus capsids for isolation of viral DNA and RNA (Fister et al., 2015).

In the presented study we conducted an overall analysis of the effects of different elements of IL structures on two model, non-enveloped viruses: *Listeria monocytogenes* phage P100 and *Escherichia coli* phage MS2. Phage P100 is commercially available as the active ingredient in Listex™ P100 (Micreos Food Safety BV, The Netherlands), an anti-*Listeria* intervention product designed to combat pathogen contamination in food processing plants. P100 is a member of the *Myoviridae*, has a 131 kilo base pair genome with dsDNA, a contractile tail, a molecular weight of about 1.2 × 10^8^ Dalton and a length of ~300 nm (Carlton et al., 2005). It is known to be stable at high sodium chloride concentrations (up to saturated solutions; EFSA, 2012). Phage MS2, a common surrogate for enteric RNA viruses, especially human noroviruses (Bae and Schwab, 2008; Mikel et al., 2016), has an isometric shape, +ssRNA, a diameter of 26 nm (Dawson et al., 2005) and is stable in monovalent salt concentrations of at least 1 M (Mylon et al., 2010). The aim of the study was to investigate if known IL structure activity relationship (SAR) outcomes can be applied to viruses or if additional or new SAR can be found. Specifically we tested the effects of cationic side chain length, the number of cationic side chains (reflecting increasing IL lipophilicity) and changing the cation head group on infectivity of the phages. In respect of the anion, we investigated the effects of increased chaotropicity, increasing the alkyl side chain lengths and the effects of halides, phosphorus and sulfur-based anions.

## Materials and methods

### Phages and host strains

Phage MS2 was used as a model for RNA viruses (kindly provided by Prof. Regina Sommer, Medical University of Vienna). Phage P100 was used as a model for DNA viruses and was purchased as Listex™ P100 solution (Batch 12G26, Lot: 308; Micreos, Wageningen, NL). For numeration and replication of phage MS2, *E. coli* Top 10F′ (Invitrogen) was used. For propagation and enumeration of phage P100, *L. monocytogenes* EGDe (ATCC BAA-679) was used. All bacteria strains were grown overnight in tryptone soya broth (TSB) with 0.6% (w/v) yeast extract (Oxoid Ltd., Hampshire, UK) at 37°C. Overnight cultures were diluted 10-fold in fresh medium and incubated at 37°C for 3–4 h to obtain a maximum number of viable cells in the logarithmic growth phase (log phase). For production of virus stocks of phage MS2, the phage solutions were used for plaque assays (see Section Ionic Liquids). The plates with confluent lysis were overlaid with 5 ml SM buffer (5.8 g NaCl, 2.4 g Tris HCl, 1.0 g CaCl_2_, 0.1 g gelatine, add. 1,000 ml H_2_O, pH 7.5) and shaken overnight at 4°C. Thereafter the SM buffer was centrifuged at 8,000 rpm for 2 min. The supernatant was filtered (0.02 μm), aliquoted and stored at −20°C.

### Ionic liquids

The influence of 55 ILs on the infectivity of phages MS2 and P100 was tested. [C_2–6_mim][Cl] and [C_4_mim][MeSO_4_, DCA, SCN and TCM] were provided by Merck KGaA (Darmstadt, Germany). [DODMA][Cl], [TMC_8,12, and 16_A][Cl], [C_4_mim][I and TCA] were synthesized using the CBILS®^1^ route (Kalb et al., 2005) as previousely described (Fister et al., 2015). Precusor ILs ([TMC_8,12, and 16_A] and [C_4_mim] [MC]) were provided by Proionic GmbH (Grambach, Austria). Iodic acid was purchased from Sigma-Aldrich Chemie GmbH (Steinheim, Germany), hydrochloric acid and trichloroacetic acid were obtained from Merck KGaA (Darmstadt, Germany). All other ILs were provided by Proionic GmbH (Grambach, Austria). A complete list of the ILs tested, including structural formulas, can be found in the Supplementary Data (Table S1).

### Experimental procedure and phage numeration

Phages, either MS2 or P100 (~10^10^ PFU/ml), were mixed with each IL (final concentrations 5 to 0.1%; solid ILs w/v; liquid ILs v/v in water) and incubated for 30 min at room temperature. Thereafter serial dilutions were prepared and the virus concentration determined by the Double Agar Overlay Plaque Assay (Kropinski et al., 2009) using log phase cultures of *L. monocytogenes* EGDe and *E. coli* TOP 10F'. The log_10_ reduction of phage concentration (plaque forming units (PFU)/ml), in comparison to the untreated control, was calculated.

All experiments were repeated on at least 3 separate days and the Double Agar Overlay Plaque Assay was performed at least with two different dilutions of the serial dilution. The detection limit was about 5–6 log_10_ reduction (depending on the ILs and their influence on the bacteria). Investigations on the influence of cations, including different alkyl side chain numbers and lengths, and testing of the effects of anions with increasing chaotropicity, were performed using both phages. All further experiments were performed using only phage P100, as this phage was the most sensitive. In the case of >2 log_10_ PFU reductions of phage P100, the experiment was repeated using phage MS2. All ILs were initially tested at a concentration of 5% (w/v) in water. To investigate if IL effects were concentration-dependent, ILs with increasing cationic alkyl side chains, which caused a PFU reduction of >2 log_10_ units, were additionally tested using IL concentrations of 2.5, 1, and 0.1%.

## Results and discussion

**Figure 1:**
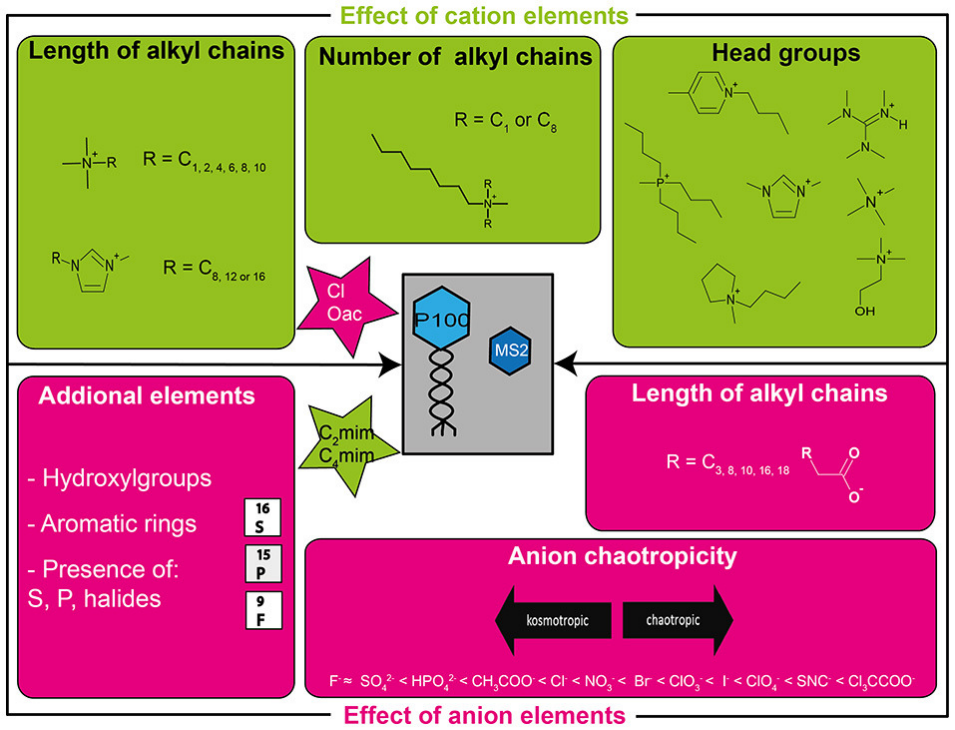
Overview of the structural elements of ILs tested in this study. Effects of cations (structures are illustrated in the fields in the upper half of the figure) were tested using ILs with chloride or acetate anions (illustrated in the upper star) and the effects of the anions (elements illustrated in the lower fields) were tested using ILs with C_2_mim or C_4_mim cations (illustrated in the lower star).

Although ILs were originally considered “green solvents,” particularly on account of their negligible vapor pressures, it has become clear that they are potentially toxic and their biodegradability questionable (Santos et al., 2015; Ławniczak et al., 2016). The toxic effects of ILs have already been described on several biological test systems and some heuristic rules based on certain structural elements of ILs have been published (summarized by Thuy Pham et al., 2010). However, since these test systems did not include viruses or phages, which nevertheless are the most abundant biological entities on the world (Clokie et al., 2011), we attempted in this study a systematic analysis to determine if known SARs are applicable to viruses and if ILs might be candidates as new potential virucidal agents. ILs tested in this study differed in several structural characteristics, such as the cation core, anion, and the length of the cationic alkyl side chains (Figure 1).

### The cationic “side chain effect” can also be observed in viruses

As most chemical and physical properties of ILs can be determined by combinations of certain structural elements at the cation and the anion, ILs are considered to be “designer solvents” (Cull et al., 2000). This tuneability can also be applied to determine the toxicity of ILs, and it has been demonstrated that the choice of the cation in particular influences IL toxicity (Ranke et al., 2004; Couling et al., 2006). It is mainly the length of the alkyl side chain of the cation that is considered responsible for increased bioaccumulation and enhanced toxicity (Ranke et al., 2007; Ventura et al., 2012; Grzonkowska et al., 2016). In this study the cationic side chain effect was investigated using imidazolium and ammonium-based cations and chloride anions (chloride anions are considered non-virucidal). Imidazolium-based ILs did not distinctly reduce both phage numbers until a side chain length of C_8_ was reached. [C_10_mim][Cl] reduced virus infectivity more than 5 log_10_ units (P100) and about 2.5 log_10_ units (MS2). Similar results were found for ammonium-based ILs (Table 1). No PFU reduction was found for [TMC_8_A]Cl, while a slight and concentration-dependent virus inactivation was found for [TMC_12_A]Cl. Phage P100 was again more sensitive than MS2 to this IL (P100: 2 log_10_ reduction vs. MS2: 1.2 log_10_ reduction). [TMC_16_A]Cl also resulted in higher reductions of phage P100 (3 log_10_ units at 5% IL) than of phage MS2 (1.3 log_10_ units). Lower concentrations did not lead to distinct differences in infectivity between the tested phages.

**Table 1:** Side chain effect of imidazolium and ammonium-based ionic liquids and the effect of the number of side chains (represented by TMC_8_A, DODMA, and TOMA).

**IL**	**Tested concentration %**	**P100 reduction**	**MS2 reduction**
**IMIDAZOLIUM-BASED ILs**			
[C_1_mim][Cl]	5	n.r.^a^	n.r.
[C_2_mim][Cl]	5	n.r.	n.r.
C_4_mim][Cl]	5	n.r.	n.r.
[C_6_mim][Cl]	5	n.r.	n.r.
[C_8_mim][Cl]	5	n.r.	n.r.
[C_10_mim][Cl]	5	>5^b^	2.48 (±1.17)^c^
	2.50	2.27 (±0.79)	1.83 (±0.51)
	1	1.15 (±1.40)	1.53 (±1.25)
	0.10	n.r.	n.r.
**AMMONIUM-BASED ILs**			
[TMC_8_A][Cl]	5	n.r.	n.r.
[TMC_12_A][Cl]	5	2.00 (±1.29)	1.24 (±1.03)
[TMC_16_A][Cl]	5	3.11 (±1.70)	1.32 (±0.51)
	2.50	1.25 (±0.86)	1.53 (±0.95)
	1	n.r.	1.22 (±1.07)
	0.10	n.r.	n.r.
[DODMA][Cl]	5	4–>5	3.03 (±0.67)
	2.50	4.40 (±0.37)	2.34 (±0.76)
	1	4.41 (±0.87)	1.98 (±0.25)
	0.10	n.r.	n.r.
[TOMA][Cl]	5	n.r.	2.83 (±0.62)
	2.50	n.r.	3.25 (±0.29)
	1	n.r.	3.26 (±0.29)
	0.10	n.r.	2.44 (±0.21)

*The table shows the reduction of P100 and MS2 after 30 min incubation in the respective ionic liquid*.

a *No distinct reduction (<1 log_10_)*.

b *Reduction was higher than the detection limit (5–6 log_10_ reduction)*.

c *Log_10_ reduction (± standard deviation)*.

*If there was >2 log_10_ reduction IL with concentrations <5% were tested*.

The side chain effect is normally explained by increased lipophilicity and surface activity of ILs with elongated side chains, which give them a surfactant-like behavior. For cellular organisms increased surfactant-like behavior leads to a non-specific disturbance of biological membranes and therefore increased toxicity (Rosen et al., 2001; Ranke et al., 2007). Similar results, for instance, have been reported by Egorova et al. (2015) and Bubalo et al. (2014). However, this explanation cannot be applied to the phages tested in this study. Non-enveloped viruses, such as phages P100 and MS2, do not possess membranes and this could explain their stability to ILs with long alkyl side chains. Observed inactivation of phages by long-chained ILs could be due to protein denaturation (as the capsid of the phages consists of proteins). However, it must be mentioned that from this particular experimental approach it is not clear if the whole virus particle is denatured or disintegrates or if only the specific binding sites for host recognition are compromised, which would prevent phage-host interaction. In another study the effect of CTAB (cetyltrimethylammoninium bromide) was tested on phages c2 and MS2. In keeping with the results of our study, here RNA phage MS2 infectivity was not reduced while infectivity of the DNA phage c2 was reduced (Chatain-Ly et al., 2013).

Our finding that IL antiviral activity is concentration-dependent is consistent with observations made elsewhere, although much lower concentration are necessary to cause toxic effects in the tested plants (0.01% IL in soil; Biczak et al., 2014, 2016). Moreover, our results demonstrate the much greater stability of non-enveloped viruses in comparison to their host bacteria (*L. monocytogenes* and *E. coli*), which are effectively killed (99.99% CFU reduction) by a ~500 times lower concentration of [C_10_mim][Cl] (Weyhing-Zerrer et al., 2017).

### The number of cationic alkyl side chains causes diverse patterns of virus reduction

In addition to ILs with a single elongated alkyl side chain, ammonium-based cations with two and three elongated alkyl side chains were tested. A previous study by Byrne et al. (2012) concluded that ammonium-based ILs with either one or two ethyl or propyl side chains tended to stabilize the tested tobacco mosaic virus while congeners with three side chains destabilized.

In the present study [DODMA][Cl] (two octyl side chains) proved to be more effective against both tested phages compared to ILs with one elongated alkyl side chain represented by [TMC_8_A][Cl] (see Table 1). PFU reductions >5 log units (5% IL), 4.4 log_10_ units (2.5 and 1%) and <1 log_10_ unit (0.1%) were obtained for phage P100, while phage MS2 was less sensitive, showing a highest reduction of 3 log_10_ units after incubation with 5% IL. This observation is not in accordance with the results of Byrne et al. (2012) since virus destabilization was observed. It is possible that destabilization results from increased lipophilicity on account also of higher number of C atoms and not only due to increasing the side chain numbers (Santos et al., 2015).

Following the trend observed by Byrne et al. (2012), we found that an ammonium-based IL with three alkyl side chains ([TOMA][Cl]) was effective against phage MS2. This congener resulted in PFU reductions of about 3 log units at all concentrations tested. Yet, it is not clear if the observed effect occurs because [TOMA] is an ammonium-based IL with three C_8_ chains or because more elongated alkyl side chains increase IL lipophilicity.

Surprisingly, and in contrast to the results with phage MS2, there was almost no effect of [TOMA][Cl] on phage P100 at any concentration tested. This was especially interesting as, with all other tested ILs, phage MS2 was more stable compared to phage P100. No ready explanation is available and further investigations with other [TOMA]-based ILs are required.

A general explanation for the different observed effects of ILs on the tested phages could relate to the different structures and sizes of the phages themselves. Phage P100 is much larger than MS2, containing a cubic head and a contractile tail comprising a sheath and a central tube. The tail could be the sensitive component of P100 as it is necessary for successful host recognition and binding (Ackermann, 2003). In contrast, polyhedral phage MS2 is smaller and has no tail and this probably enhances its stability against most ILs. However, the different structures and sizes do not explain all the effects that were observed.

### The cationic head group has no influence on virus infectivity

Although the side chain lengths of cations are considered to be the main modulator of IL toxicity, the head group also contributes to toxicity. Aromatic cations have higher solubility in water and are more toxic (Freire et al., 2007; Ventura et al., 2013). In general, IL toxicity is expected to be lowest with ammonium, followed by pyrimidinium and imidazolium-based ILs (Couling et al., 2006; Stolte et al., 2007). In this study we tested seven different head groups (Table 2). Additionally, we included a second ammonium-based IL with three butyl-side chains in order to exclude the possibility that the reduction of phage infectivity by [TBMP] is associated with the length or number of cationic alkyl side chains. We found that the phosphonium-based IL [TBMP][Oac] reduced virus infectivity by about 2 log_10_ units, while all other tested head groups do not influence phage stability.

**Table 2:** Effect of cationic head group.

**IL**	**Log_10_ reduction**
[C_2_MIM][Oac]	n.r.^a^
[TMA][Oac]	n.r.
[TBMA][Oac]	n.r.
[TBMP][Oac]	1.50 (±1.55)^b^
[1,1,2,3,4, Pentamethylguanidinium] [Oac]	n.r.
[EMMor][Oac]	n.r.
[Cholinium][Oac]	n.r.
[BMPyr][Br]	n.r.

*Reduction of P100 in 5% ionic liquid after 30 min incubation*.

a *No distinct reduction (<1 log_10_)*.

b *Log_10_ reduction (± standard deviation)*.

### No heuristic rules describing the effect of anions on viruses could be found

Studies of IL toxicity in cellular organisms began with the finding that the anion moiety plays a minor role (Stolte et al., 2006). Perhaps for this reason the anion has been less well-investigated compared with the cation. However, more detailed investigations led to the conclusion that toxicity was not limited to cation effects and especially in ILs with short-chained cations also the anion influences the toxicity of ILs (Stolte et al., 2007).

We previously showed that anion chaotropicity is a major factor that influences the antimicrobial activity of ILs with cation side chain lengths ≤6 (Mester et al., 2012). ILs equipped with chaotropic anions could therefore be promising candidates for virus inactivation. Virus protective capsids comprise protein units and these should be susceptible to denaturation. We used ILs with the non-virucidal cation [C_4_mim] and anions representing the Hofmeister series to test this hypothesis. Results presented in Table 3 show that even strong chaotropic anions, such as trichloroacetate or thiocyanate, did not lead to significant PFU reductions. In other words neither phage was inactivated. Also surprising was that iodine-containing ILs had no significant effect on the tested viruses, which were still infective after 30 min of IL incubation, even although iodine is known to inactivate phage MS2 rapidly (Brion and Silverstein, 1999).

**Table 3:** Effect of anions with increasing chaotropicity expressed by the reduction of infectivity (log_10_) of phages P100 and MS2 after 30 min incubation in 5% IL.

**IL**	**P100 reduction**	**MS2 reduction**
[C_4_mim][MeSO_4_]	n.r.^a^	n.r.
[C_4_mim][I]	n.r.	1.04 (± 0.17)^b^
[C_4_mim][DCA]	n.r.	n.r.
[C_4_mim][SCN]	n.r.	n.r.
[C_4_mim][TCM]	n.r.	n.r.
[C_4_mim][TCA]	n.r.	n.r.

a *No distinct reduction (<1 log_10_)*.

b *log_10_ reduction (± standard deviation)*.

General toxicity of ILs cannot be restricted to chaotropic anions. Ventura et al. (2014) showed that IL toxicity not only resulted from elongated cation side chains, but also from the anion. In our study we compared the effect of [C_2_mim][Propionate] (C_3_), [Caprylate] (C_8_), [Caprynate] (C_10_), [Palmitate](C_16_) and [Stearate](C_18_) on phage P100 and observed a reduction of about 4 log_10_ units. [C_2_mim][Caprynate] and [Stearate] resulted in 2 log_10_ unit reductions (Table 4, structure and IUPAC names are shown in Table S2). The other ILs did not distinctly effect P100. [C_2_mim][Oxalate] caused a reduction of phage P100 of nearly 3 log_10_ units. Moreover, all of the tested ILs with increasing lengths of anion alkyl side chains, which caused a reduction of P100 infectivity, did not distinctly reduce the infectivity of phage MS2 (Table 5). Although, some of the anions with longer side chains were active against P100, no clear trend as shown for the cationic side chain effect was found. Therefore, a “side chain-like” effect of the anion on viruses cannot be confirmed. These results contrast with those of Ventura et al. (2014) who suggested that long anion alkyl chains have a higher toxicity to *Vibrio fischeri* and marine bacteria. However, inconsistency in IL toxicity across different organisms has also been observed elsewhere (Matzke et al., 2007; Stolte et al., 2007; Egorova and Ananikov, 2014).

**Table 4:** Effects of alkyl chain length, OH groups, and anion aromatic rings.

**IL**	**Log_10_ reduction**
[C_2_mim][Oxalate]	3.98 (±0.30)^a^
[C_2_mim][Propionate]	n.r.^b^
[C_2_mim][Malonate]	n.r.
[C_2_mim][Methoxyacetate]	n.r.
[C_2_mim][Lactate]	n.r.
[C_2_mim][Pyruvate]	n.r.
[C_2_mim][Pivalate]	n.r.
[C_2_mim][Benzoate]	n.r.
[C_2_mim][Salicylate]	3.38 (±0.45)
[C_2_mim][Caprylate]	n.r.
[C_2_mim][Caprynate]	4.32 (±0.29)
[C_2_mim][Palmitate]	n.r.
[C_2_mim][Stearate]	2.10 (±1.86)

*Reduction of P100 after 30 min incubation in 5% of the respective ionic liquid*.

a *Log_10_ reduction (± standard deviation)*.

b *No distinct reduction (<1 log_10_)*.

**Table 5:** Reduction of MS2 infectivity by ILs with carboxylate anions, [NH_2_SO_3_]− or [FeCl_4_]−after 30 min incubation in 5% IL.

**IL**	**Log_10_ reduction**
[C_2_mim] [Oxalate]	n.r.^a^
[C_2_mim][Salicylate]	n.r.
[C_2_mim][Caprynate]	n.r.
[C_2_mim][Stearate]	n.r.
[C_2_mim][NH_2_SO_3_]	n.r.
[C_2_mim][FeCl_4_]	1.93 (±0.22)^b^

a *No distinct reduction (<1 log_10_)*.

b *Log_10_ reduction (±standard deviation)*.

We also investigated if introductions of aromatic rings, hydroxyl groups, or oxygenation change the effect of ILs on virus infectivity. Santos et al. (2015) reported that in cholinium-based ILs the introduction of a single hydroxyl group did not change IL toxicity, while the addition of three hydroxyl groups increased polarity and toxicity. Nevertheless, cholinium-based ILs do behave differently than other ILs, especially in respect of the side chain effect and because they have an increased bioavailability Santos et al. (2015). In contrast, oxygenation via carboxylic addition did decrease cholinium-based IL toxicity. In the present study no change in P100 infectivity was observed when different structured C_3_ carboxylates were tested (Table 4). When testing [C_2_min][Benzoate] and [Salicylate], which differ only in one hydroxyl group, [Benzoate] resulted in only a 0.18 log_10_ reduction of P100 infectivity, while [Salicylate] reduced phage P100 infectivity by 3.38 log_10_ units (Table 4). On one hand these results indicate that in general aromatic rings at the anion do not seem to influence P100 stability. This was also found by Ventura and colleagues, who did not discern a clear toxic tendency of aromatic anions (Ventura et al., 2014). On the other hand one could speculate from these results that the higher reduction caused by [C_2_mim][Salicylate] might be due to the additional OH group. The data does not permit further insight. Interestingly all tested anions based on carbonic acids that caused a P100 phage reduction of >2 log_10_ units phage did not cause a distinct reduction of MS2 (Table 5). From the data it can be concluded that different structures of carboxylate anions do not influence virus infectivity. However, the generality of this statement must await further studies using more and different anions and additional viruses.

In addition to the different carboxylate anions tested, we also examined the phage P100 effects of anions based on sulfur and phosphorus. With the exception of [C_2_mim][NH_2_SO_3_], which reduced P100 infectivity by 4 log_10_ units, the five tested sulfur and five tested phosphorus-based anions (Tables 6, 7) had no effects of P100. As observed previously, [C_2_mim][NH_2_SO_3_], which caused a significant reduction in P100 numbers, had no effect on MS2 infectivity (Table 5), attesting to the higher stability of this phage.

**Table 6:** Reduction of phage P100 infectivity by sulfur-containing anions after 30 min incubation in 5% IL.

**IL**	**Log_10_reduction**
[C_2_mim][EtSO_4_]	n.r.^a^
[C_2_mim][NH_2_SO_3_]	4.05 (±2.05)^b^
[C_2_mim][MESO_3_]	n.r.
[C_2_mim][HOCH_2_SO_3_]	n.r.
[C_2_mim][Tetrathiomolybdate]	n.r.

a *No distinct reduction (<1 log_10_)*.

b *Log_10_ reduction (± standard deviation)*.

**Table 7:** Reduction of P100 infectivity by phosphorus-containing anions after 30 min incubation in 5% IL.

**IL**	**Log_10_ reduction**
[C_2_mim][DEP]	n.r.
[C_2_mim][DMP]	n.r.
[C_2_mim][H_2_PO_4_]	n.r.
[C_2_mim][DBP]	1.20 (±0.62)
[C_2_mim][TDecHPO_3_]	n.r.

*^a^No distinct reduction (<1 log_10_)*.

*^b^Log_10_ reduction (± standard deviation)*.

We also examined the influence of halide anions on phage P100. Altogether six different fluorine-containing anions were compared. This included [C_2_mim][NtF_2_] (Table 8), which has already been shown to exert toxicity to several biological test systems (Matzke et al., 2007). However, in our investigation none of the tested anions reduced P100 infectivity.

**Table 8:** Reduction of phage P100 infectivity by halide anions after 30 min incubation in 5% IL.

**IL**	**Log_10_ reduction**
[C_2_mim][Tris(pentafluoroethyl)triflurophosphate]	n.r.
[C_2_mim][Heptafluorobutonate]	n.r.
[C_2_mim][Bis(trifluoromethylsulfonyl)imide]	n.r.
[C_2_mim][Trifluoromethanesulfonate]	n.r.
[C_2_mim][Heptafluorotantalate]	n.r.
[C_2_mim][Trifluoroacetate]	n.r.
[C_2_mim][FeCl_4_]	>5

*^a^No distinct reduction (<1 log_10_)*.

*^b^Log_10_ reduction (±standard deviation)*.

*>5: reduction was higher than the detection limit indicating >5 log_10_ reduction*.

The paramagnetic IL [C_2_mim][FeCl_4_] was similarly investigated. It reduced P100 infectivity by about 4 log_10_ (Table 8) and MS2 infectivity by nearly 2 log_10_ units (Table 5). Thus, FeCl_4_ was, with the exception of [DODMA][Cl], the only tested anion that distinctly reduced the infectivity of both phages. However, since a 5% solution of [C_2_mim][FeCl_4_] has a pH value of about one, the pronounced pH change likely accounted for the reductions. We have already shown that phage P100 infectivity rapidly decreases when it is incubated at pH values less than two (Fister et al., 2016). Another reason for infectivity reduction could be aggregation and virus particle precipitation. For example, divalent ions are known to enhance aggregation and FeCl_3_, which has been used to precipitate viruses from sea water, is known to induce virus flocculation (Gutierrez et al., 2010; Da Silva et al., 2011; John et al., 2011).

In conclusion, the aim of the study was to examine the effects of ionic liquids in terms of structure-activity relationships on the infectivity of viruses. While diverse patterns of infectivity reductions were obtained for each phage, mostly phage P100 was more sensitive than phage MS2. With the exception of the cationic “side chain effect” that could be observed with both tested viruses, other known SARs were not applicable to the tested viruses and no heuristic rules are as yet possible. None of the tested ILs could be readily classified as virucidal against both viruses at the concentrations tested.

Overall, this study reveals that in respect of IL toxicity, results from eukaryotes or prokaryotes are not transferrable to viruses. This significantly limits the usefulness of previously published data in terms of future antiviral applications. Our results highlight the need of further systematic studies identifying antiviral IL-SARs, yet offer a considerable basis for such studies.

## Author contributions

Conception and design of the work: SF, PM, MW, PR; Acquisition of data: SF, JS, Analysis and interpretation of data: PM, SF, AW, JS, RK; Drafting the work: SF, PM, AW; Revision of the manuscript: SF, PM, AW, RK, MW, PR. All authors approved the version to be published in Frontiers in Microbiology and agreed to be accountable for all aspects of the work.

### Conflict of interest statement

The authors declare that the research was conducted in the absence of any commercial or financial relationships that could be construed as a potential conflict of interest.

## Footnotes

^1^CBILS® is a registered trademark of Proionic GmbH.
