# Supplementary material for: Virucidal Influence of Ionic Liquids on Phages P100 and MS2
Source: Front Microbiol. 2017 Aug 24;8:1608. doi: 10.3389/fmicb.2017.01608 (PMC5573800; doi:10.3389/fmicb.2017.01608)
Supplement: Supplementary file 1 [file Table1.DOCX]

Supplemental Tables

| **Table S1**  **Names and structures of all tested ILs** | | |
| --- | --- | --- |
| Tested ILs | Cation | Anion |
| [1,1,2,3,4, Pentamethylguanidinium][Oac] |  | (Acetate) |
| [BMPyr][Br] |   N-Butyl-N-methyl-pyrrdidinium | Br^-^  (Bromide) |
| [C_10_mim][Cl] |  | Cl^-^  (Chloride) |
| [C_1_mim][Cl] |  | Cl^-^  Chloride |
| [C_2_mim][Benzoate] |   1-Ethyl-3-methylimidazolium |  |
| [C_2_mim][Bis(trifluoromethylsulfonyl)imide] |  |  |
| [C_2_mim][Caprylate] |  |  |
| [C_2_mim][Caprynate] |  |  |
| [C_2_mim][Cl] |  | Cl^-^ (Chloride) |
| [C_2_mim][DBP] |  |   Dibutylphosphate |
| [C_2_mim][DEP] |  |   Diethylphosphate |
| [C_2_mim][DMP] |  |   Dimethylphosphate |
| [C_2_mim][EtSO4] |  |   Ethylsulfate |
| [C_2_mim][FeCl4] |  | Irontetrachloride |
| [C_2_mim][H2PO4] |  |   Dihydrogenphosphate |
| [C_2_mim][Heptafluorobutonate] |  |  |
| [C_2_mim][Heptafluorotantalate] |  |  |
| [C_2_mim][HOCH_2_SO_3_] |  |   Hydroxymethanesulfonate |
| [C_2_mim][Lactate] |  |  |
| [C_2_mim][Malonate] |  |  |
| [C_2_mim][MeSO_3_] |  |   Methanesulfonate |
| [C_2_mim][Methoxyacetate] |  |  |
| [C_2_mim][NH_2_SO_3_] |  | Ammoniumsulfate |
| [C_2_MIM][Oac] |  | Acetate |
| [C_2_mim][Oxalate] |  |  |
| [C_2_mim][Palmitate] |  |  |
| [C_2_mim][Pivalate] |  |  |
| [C_2_mim][Propionate] |  |  |
| [C_2_mim][Pyruvate] |  |  |
| [C_2_mim][Salicylate] |  |  |
| [C_2_mim][Stearate] |  |  |
| [C_2_mim][TDecHPO_3_] |  | Tridecylhydrogenphosphate |
| [C_2_mim][Tetrathiomolybdate] |  |  |
| [C_2_mim][Trifluoroacetate] |  |  |
| [C_2_mim][Trifluoromethanesulfonate] |  |  |
| [C_2_mim][Tris(pentafluoroethyl)triflurophosphate] |  |  |
| [C_4_mim][Cl] |   1-Butyl-3-methylimidazolium | Cl^-^  Chloride |
| [C_4_mim][DCA] |  |   Dicyaneamide |
| [C_4_mim][I] |  | I-  Iodid |
| [C_4_mim][MeSO_4_] |  | Methylsulfate |
| [C_4_mim][SCN] |  |   Thiocyanate |
| [C_4_mim][TCA] |  | Trichloracetate |
| [C_4_mim][TCM] |  | Tricyanomethanide |
| [C_6_mim][Cl] |   1-Hexyl-3-Methylimidazolium | Cl^-^  Chloride |
| [C_8_mim][Cl] |   1-Octyl-3-Methylimidazolium |  |
| [Cholinium][Oac] |  | Acetate |
| [DODMA][Cl] |   Dioctyldimethylammonium | Cl^-^  Chloride |
| [EMMor][Oac] |   Ethyl-Methylmorpholinium | Acetate |
| [TBMA][Oac] |   Tributylmethylammonium |  |
| [TBMP][Oac] |   Tributylmethylphosphonium |  |
| [TMA][Oac] |   Tetramethylammonium |  |
| [TMC_12_A][Cl] | Trimethyldodecylammonium | Cl^-^  Chloride |
| [TMC_16_A][Cl] |   Trimethylhexylammonium |  |
| [TMC_8_A][Cl] |   Trimethyoctylammonium |  |
| [TOMA][Cl] |   Trioctylmethylammonium |  |

| **Table S2**  Molecular and structural formula and IUPAC name of carbonic acid based anions | | | | |
| --- | --- | --- | --- | --- |
| **IL** | **Molecular**  **Formula** | ***IUPAC Name*** | **Structural formula** |  |
| [C_2_mim][Oxalate] | C_2_HO_4_ | Oxalate | 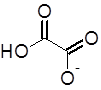 | \|  \| \| --- \| |
|  |  |  | 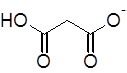 |  |
| [C_2_mim][Malonate] | C_3_H_3_O_4_ | Malonate |  |  |
|  |  |  | 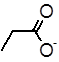 |  |
| [C_2_mim][Propionate] | C_3_H_5_O_2_ | Propionate |  |  |
|  |  |  | 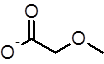 |  |
| [C_2_mim][Methoxyacetate] | C_3_H_5_O_3_ | Methoxyacetate |  |  |
|  |  |  | 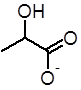 |  |
| [C_2_mim][Lactate] | C_3_H_5_O_3_ | 2-Hydroxy  propanoate |  |  |
|  |  |  | 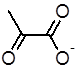 |  |
| [C_2_mim][Pyruvate] | C_3_H_3_O_3_ | 2-Oxopropanoate |  |  |
|  |  |  | 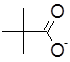 |  |
| [C_2_mim][Pivalate] | C_5_H_9_O_2_ | Pivalate |  |  |
|  |  |  |  |  |
| [C_2_mim][ Benzoate] | C_7_H_5_O_2_ | Benzoate | 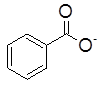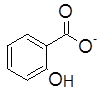 |  |
|  |  |  |  |  |
| [C_2_mim][Salicylate] | C_7_H_5_O_3_ | Salicylate |  |  |
|  |  |  |  |  |
| [C_2_mim][Caprylate] | C_8_H_15_O_2_ | Octanoate | 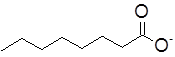 |  |
|  |  |  | 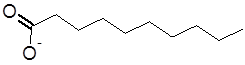 |  |
| [C_2_mim][Caprynate] | C_10_H_19_O_2_ | Decanoate |  |  |
|  |  |  | 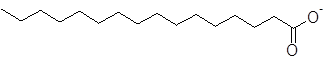 |  |
| [C_2_mim][Palmitate] | C_16_H_31_O_2_ | Palmitate |  |  |
|  |  | 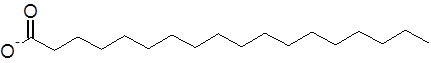 |  |  |
| [C_2_mim][Stearate] | C_18_H_35_O_2_ | Sterate |  |  |
|  |  |  |  |  |
